# Antifungal, antiaflatoxigenic, and cytotoxic properties of bioactive secondary metabolites derived from *Bacillus* species

**DOI:** 10.1038/s41598-024-66700-y

**Published:** 2024-07-18

**Authors:** Aya Abdel-Nasser, Ahmed N. Badr, Hayam M. Fathy, Mosad A. Ghareeb, Olfat S. Barakat, Amal S. Hathout

**Affiliations:** 1 Food Toxicology and Contaminants Department, Food Industry and Nutrition Research Institute, National Research Centre, Dokki, Cairo, 12622 Egypt; 2 Agricultural Microbiology Department, Faculty of Agriculture, Cairo University, Giza, Egypt; 3 Medicinal Chemistry Department, Theodor Bilharz Research Institute, Kornaish El Nile, Warrak El-Haddar, Imbaba, (P.O. 30), Giza, 12411 Egypt

**Keywords:** *Bacillus* species, Bioactive metabolites, Antifungal activity, Antiaflatoxigenic activity, Brine shrimp bioassay, Hepatocellular carcinoma, Biotechnology, Cancer, Microbiology

## Abstract

Aflatoxins (AFs) are hazardous carcinogens and mutagens produced by some molds, particularly *Aspergillus* spp. Therefore, the purpose of this study was to isolate and identify endophytic bacteria, extract and characterize their bioactive metabolites, and evaluate their antifungal, antiaflatoxigenic, and cytotoxic efficacy against brine shrimp (*Artemia salina*) and hepatocellular carcinoma (HepG2). Among the 36 bacterial strains isolated, ten bacterial isolates showed high antifungal activity, and thus were identified using biochemical parameters and MALDI-TOF MS. Bioactive metabolites were extracted from two bacterial isolates, and studied for their antifungal activity. The bioactive metabolites (No. 4, and 5) extracted from *Bacillus cereus* DSM 31T DSM, exhibited strong antifungal capabilities, and generated volatile organic compounds (VOCs) and polyphenols. The major VOCs were butanoic acid, 2-methyl, and 9,12-Octadecadienoic acid (Z,Z) in extracts No. 4, and 5 respectively. Cinnamic acid and 3,4-dihydroxybenzoic acid were the most abundant phenolic acids in extracts No. 4, and 5 respectively. These bioactive metabolites had antifungal efficiency against *A. flavus* and caused morphological alterations in fungal conidiophores and conidiospores. Data also indicated that both extracts No. 4, and 5 reduced AFB_1_ production by 99.98%. On assessing the toxicity of bioactive metabolites on *A. salina* the IC_50_ recorded 275 and 300 µg/mL, for extracts No. 4, and 5 respectively. Meanwhile, the effect of these extracts on HepG2 revealed that the IC_50_ of extract No. 5 recorded 79.4 µg/mL, whereas No. 4 showed no cytotoxic activity. It could be concluded that bioactive metabolites derived from *Bacillus* species showed antifungal and anti-aflatoxigenic activities, indicating their potential use in food safety.

## Introduction

Fungi can damage agricultural commodities during harvest, transport, and storage, resulting in decreased production and value^1,2^. Filamentous fungi are the most harmful microorganisms to agricultural products' quality and safety^3^.

Mycotoxins produced by filamentous fungi have major economic impacts on crops such as cereals, almonds, tea, pistachios, and cotton seeds^4^. Cereals and their products are an essential source of nutrients for human consumption in developing and developed countries and are considered one of the world's most critical food and feedstuffs^5^. Most cereals are susceptible to various fungal invasions during pre- and post-harvest and storage, resulting in massive yield loss, reduced seed quality, nutritional values, and germination^6,7^.

Many filamentous fungi, viz., *Alternaria, Aspergillus, Fusarium*, and *Penicillium* species*,* are primarily attached to cereal grains, making them unsuitable for human consumption by impeding their nutrient content followed by the secreting number of toxic secondary metabolites called mycotoxins^8^. As stated by the Food and Agricultural Organization of the United Nations (FAO), approximately 25% of the world's food grains are contaminated with mycotoxins at levels more remarkable than the prescribed level^9^.

Aflatoxins (AFs) represent the most widespread and common mycotoxins in food and feed, and they are primarily produced by species belonging to the *Aspergillus* section *Flavi*: *A. flavus*, *A. parasiticus*, and *A. nomius*^10,11^, whereas these fungi are considered a predominant contaminant in food grains, mainly maize^12^. Aflatoxin B_1_ (AFB_1_) is the most destructive of the known AFs, and it has been classified as a Group I human carcinogen by the International Agency for Research on Cancer^13^.

For many decades, synthetic antifungal agents or preservatives have been used to control fungal pathogens and mycotoxin contamination in foodstuffs, but some commonly used synthetic chemicals have proved to be hazardous to consumers and the environment^1,14^. To ensure consumer safety, additional control strategies that are effective, non-hazardous, and environmentally friendly are required. As a result, working to develop (micro) biological detoxifying techniques to enhance the safety of these foods for direct utilization is vital. Consequently, the biological control of toxigenic fungi and mycotoxins is regarded as one of the most contemporary means. Various microorganisms have been identified to eliminate or break down mycotoxins in food and feed. These include *Bacillus* spp.^15,16^, *Brevibacterium* spp.^17^, *Eubacterium* spp.^18^, *Flavobacterium aurantiacum*^19^, *Rhodococcus erythropolis*^20^, *Saccharomyces cerevisiae*^21^, lactic acid bacteria^22^, and others^23^. According to Schallmey et al.^24^, *Bacillus* spp. were extensively evaluated as biological agents, possibly because they proliferated, produced a wide range of antimicrobial compounds, and were generally regarded as safe (GRAS). The antimicrobial compounds produced by *Bacillus* spp. include lipopeptides, protease antibiotics, and bacteriocin^25^. *Bacillus* spp. are also known to produce bioactive secondary metabolites including polypeptides, macro-lactones, fatty acids, polyketides, and isocoumarins, and they have a broad range of biological capabilities, including antibacterial, anticancer, and anti-algal properties^26^.

Bioactive secondary metabolites are useful natural chemicals generated by numerous microorganisms, many of which have strong bioactive characteristics in the biological control of aflatoxin-producing fungi^27^. They are expelled after primary growth and throughout the stationary phase, and are among the most commercially significant industrial products and are of great interest. Bioactive metabolites, now dubbed specialized metabolites, frequently have odd structures and have shown substantial implications on the health, nutrition, and economy of our society^28^. Approximately 53% of the FDA-approved natural products-based medicines originated from microorganisms^29^.

In two previous studies, bioactive metabolites were extracted from lactic acid bacteria^30^, and *Saccharomyces cerevisiae*^31^. Therefore, the goal of this study was to isolate and identify endophytic bacteria, extract and characterize bioactive secondary metabolites, and assess their antifungal, antiaflatoxigenic, and cytotoxic activity against *A. salina* and hepatocellular carcinoma.

## Results

### Antifungal activity of cell-free supernatant (CFS) of bacterial isolates

Thirty-six bacterial isolates were isolated from rice grains. The CFS of ten bacterial isolates showed variable degrees of antifungal activity (Table 1). Results revealed that CFS of the bacteria No. RQ1 and RQ16 showed antifungal activity against *A. flavus* with a zone inhibition of 12.0, and 13.0 mm respectively. Data also showed that CFS of the bacteria No. RQ1 and RQ16 inhibited *A. parasiticus* with a zone inhibition of 20.0 mm, for both bacterial isolates. Meanwhile, the CFS of bacteria No. RQ2 showed antifungal activity against *Fusarium* spp., *A. flavus,* and *A. parasiticus* with a zone inhibition of 20, 11, and 17.0 mm respectively*.*

**Table 1:** Antifungal activity of bacterial isolates.

Fungal species	Bacterial isolates									
	RQ1	RQ2	RQ5	RQ7	RQ8	RQ13	RQ14	RQ15	RQ16	RQ22
*Aspergillus flavus*	12.00 ± 0.0	11.00 ± 0.0	14.00 ± 0.0	ND	ND	ND	ND	11.00 ± 1.4	13.00 ± 0.0	20.00 ± 0.0
*Aspergillus parasiticus*	20.00 ± 0.0	17.00 ± 0.0	ND	20.00 ± 0.0	ND	21.00 ± 5.6	ND	13.00 ± 2.0	20.00 ± 0.0	27.00 ± 2.6
*Aspergillus niger*	ND	ND	ND	13.00 ± 2.1	13.67 ± 4.0	15.00 ± 0.0	ND	15.67 ± 3.2	ND	20.00 ± 2.0
*Aspergillus ochraceus*	ND	ND	ND	ND	20.67 ± 8.3	22.50 ± 3.5	ND	ND	ND	ND
*Fusarium* spp.	ND	20.00 ± 0.0	ND	16.00 ± 0.0	ND	23.50 ± 2.1	11.00 ± 1.4	ND	ND	29.00 ± 1.4

Results are mean ± SD (*n* = 3); ND, Not detected.

R, Rice; Q, Qalyubia.

Data showed that CFS of bacteria No. RQ5 and RQ14 exhibited lower antifungal activity and inhibited one fungal species. On the other hand, the CFS of bacteria No. RQ7 and RQ15 inhibited three fungal species only. The CFS of bacteria No. RQ13 inhibited *A. parasiticus*, *A. niger*, *A. ochraceus*, and *Fusarium* spp. by 21.0, 15.0, 22.5, and 23.5 mm, respectively. Meanwhile, CFS of bacteria No. RQ22 inhibited *A. flavus*, *A. parasiticus*, *A. niger*, and *Fusarium* spp. by 20.0, 27.0, 20.0, and 29.0 mm, respectively. It was clear that the CFS of the bacteria No. RQ13 and RQ22 exhibited higher antifungal activity than other bacterial isolates. Therefore, ten bacterial isolates No. RQ1, RQ2, RQ5, RQ7, RQ8, RQ13, RQ14, RQ15, RQ16, and RQ22 showing antifungal activity were selected for their identification using biochemical analyses and MALDI TOF.

### Identification of bacterial isolates

**Figure 1:**
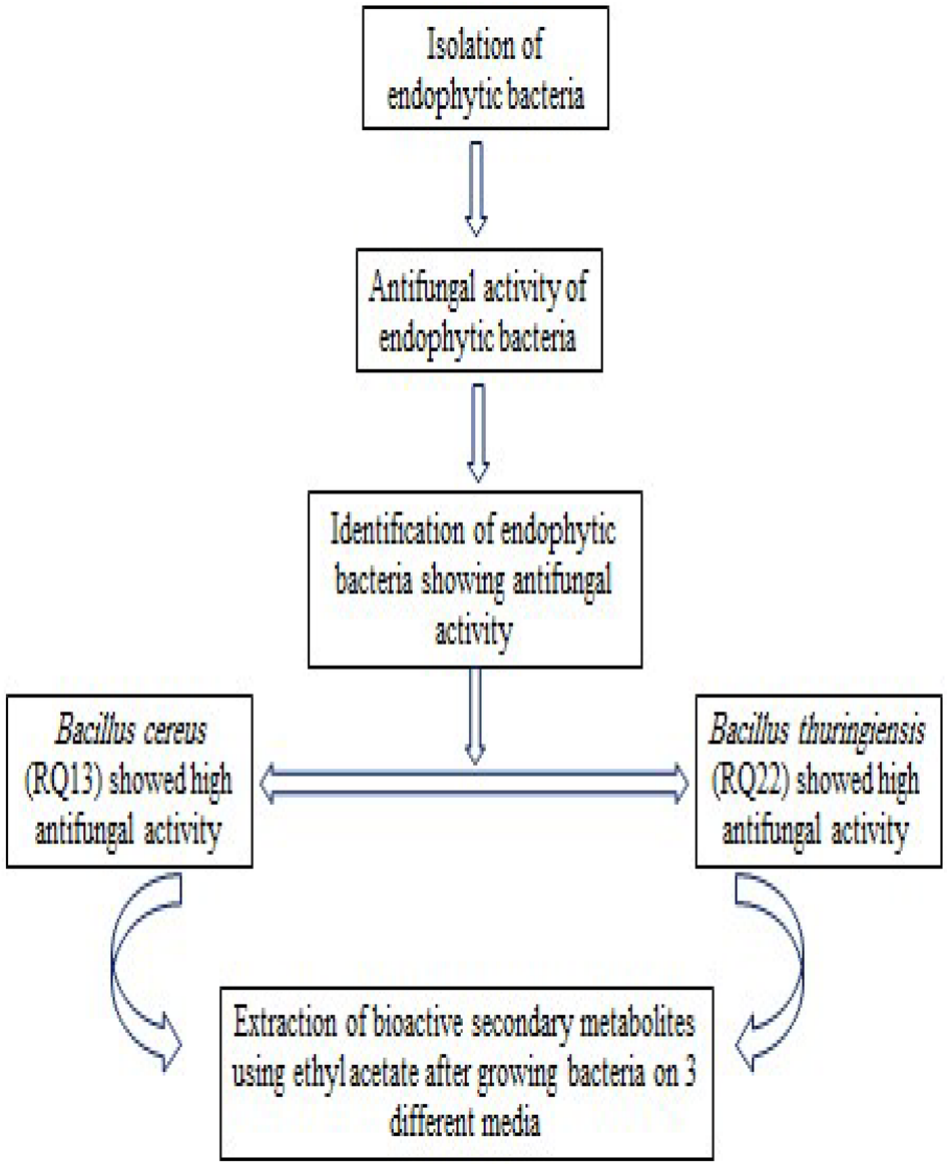
Flow chart showing the selection of suitable bacterial isolates that showed high antifungal activity for the extraction of bioactive metabolites.

**Figure 2:**
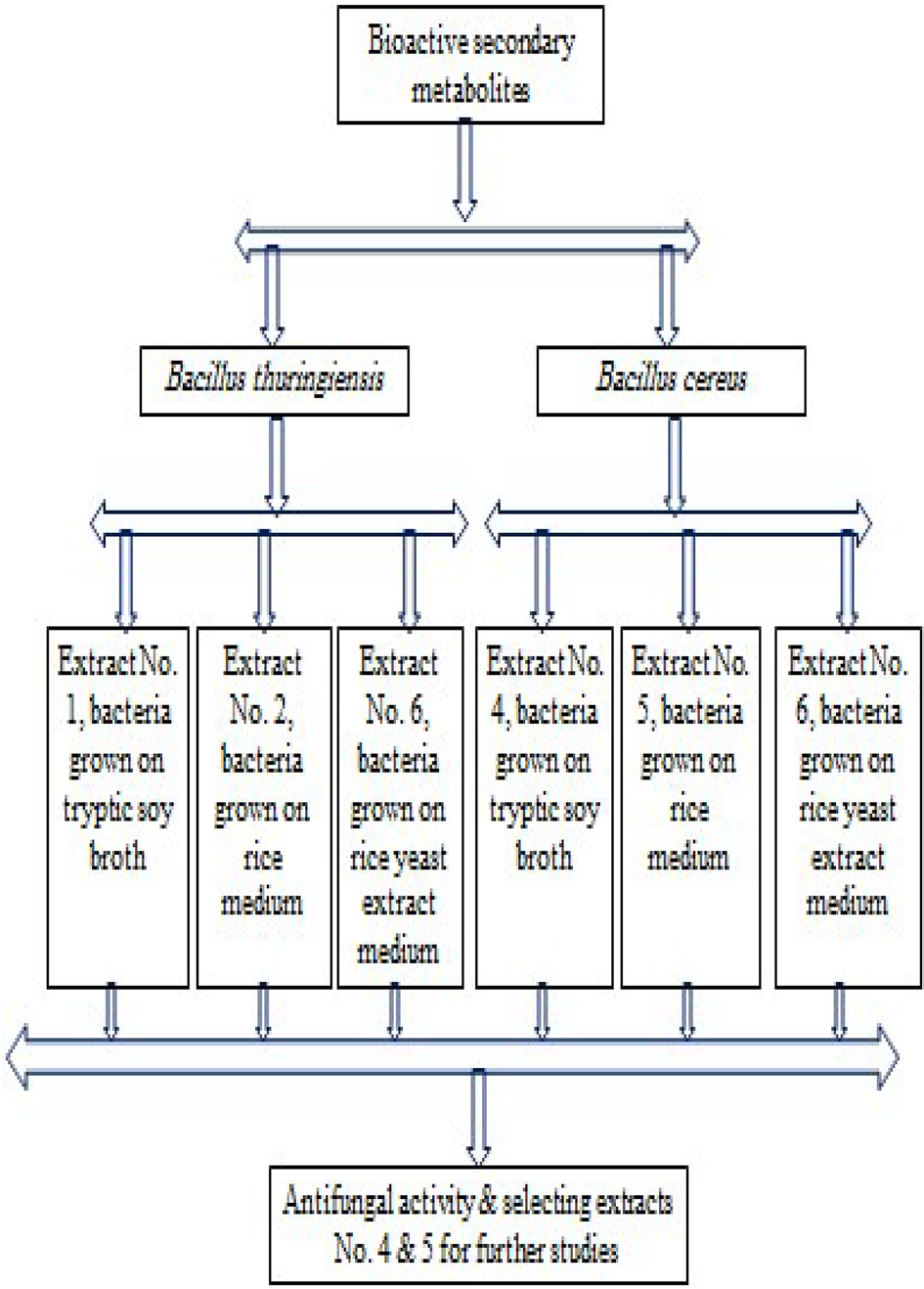
Flow chart showing the selection of suitable extracts that showed high antifungal activity.

The isolated bacteria No. RQ1, RQ2, RQ5, RQ7, RQ8, RQ13, RQ14, RQ15, RQ16, and RQ22 were identified following biochemical analyses (Table 2). Cell morphology examination and biochemical analysis revealed that all bacteria were rod-shaped (bacilli), gram-positive, and oxidase-negative. Data in Table 2 showed the MALDI-TOF MS result scores of the bacterial isolates showing antifungal activity, whereas the bacterial isolates No. RQ1, RQ5, RQ8, RQ13, and RQ16 were identified as *Bacillus cereus* DSM 31T DSM, with NCBI identifier 1396. The bacterial isolates No. RQ2, RQ7, and RQ15 were identified as *Bacillus cereus* with NCBI identifier 1396. On the other hand, the bacterial isolates RQ14 and RQ22 were identified as *Bacillus thuringiensis* DSM 2046T DSM with NCBI identifier 1428. The bacterial isolates No. RQ13 and RQ22 showing high antifungal activity were selected for the extraction of bioactive metabolites. A flow chart explaining the selection of bacterial isolates is shown in Fig. 1. Bioactive secondary metabolites were extracted from *Bacillus thuringiensis* (No. RQ22) (extracts No. 1, 2, and 3) and from *Bacillus cereus* (No. RQ13) (extracts No. 4, 5, and 6). A flow chart explaining the selection of extracts is shown in Fig. 2.

**Table 2:** Biochemical characteristics and MALDI-TOF MS result scores of bacterial isolates showing antifungal activity.

Bacterial isolates	Cell morphology	Gram reaction	Oxidase	MALDI-TOF MS score value	NCBI identifier	Identification
RQ1	Bacilli	+	−	2.24	1396	*Bacillus cereus* DSM 31 T DSM
RQ2	Bacilli	+	−	1.96	1396	*Bacillus cereus*
RQ5	Bacilli	+	−	2.01	1396	*Bacillus cereus* DSM 31 T DSM
RQ7	Bacilli	+	−	1.45	1396	*Bacillus cereus*
RQ8	Bacilli	+	−	1.97	1396	*Bacillus cereus* DSM 31 T DSM
RQ13	Bacilli	+	−	1.97	1396	Bacillus cereus DSM 31 T DSM
RQ14	Bacilli	+	−	1.60	1428	*Bacillus thuringiensis* DSM 2046 T DSM
RQ15	Bacilli	+	−	1.46	1396	*Bacillus cereus*
RQ16	Bacilli	+	−	2.13	1396	*Bacillus cereus* DSM 31 T DSM
RQ22	Bacilli	+	−	1.98	1428	*Bacillus thuringiensis* DSM 2046 T DSM

Range 2.00–3.00: High confidence and secure Genus and species Identification.

Range 1.70–1.99: Secure Genus Identification with low-confidence species identification.

Range 0.00 -1.69: Not reliable identification.

### Antifungal activity of bioactive metabolites

The six extracts of the two bacterial isolates, *Bacillus cereus* (No. RQ13) and *Bacillus thuringiensis* (No. RQ22) were used to investigate the antifungal activity against different fungal species (Table 3). Results showed that extract No. 4 exhibited the highest antifungal activity against *A. flavus*, *A. parasiticus*, *A. niger*, *A. ochraceus*, *F. oxysporum*, *Fusarium* spp., and *Penicillium* spp., recording a zone inhibition of 11.00, 12.33, 11.67, 20.00, 16.00, 11.67, and 19.00 mm, respectively. On the other hand, extract No. 5 exhibited antifungal activity against *A. flavus*, *A. parasiticus*, *A. niger*, *A. ochraceus*, and *Penicillium* spp. with a zone inhibition of 21.00, 9.33, 16.67, 15.67, and 19.00 mm, respectively. Data also revealed that extract No. 4 inhibited *F. oxysporum*, whereas other extracts could not inhibit *F. oxysporum*. Thus, extracts No. 4 and 5 were selected for further analysis, as they showed the highest antifungal activity.

**Table 3:** Antifungal activity of bioactive metabolites produced by *Bacillus* spp.

Fungal isolates	*B. thuringiensis*			*B. cereus*		
	1	2	3	4	5	6
*Aspergillus flavus*	11.67 ± 0.58	18.33 ± 16.07	ND	11.00 ± 9.64	21.00 ± 1.73	6.67 ± 11.55
*Aspergillus parasiticus*	9.33 ± 8.14	ND	16.33 ± 2.89	12.33 ± 2.08	9.33 ± 10.07	2.67 ± 4.62
*Aspergillus niger*	12.33 ± 1.53	13.33 ± 2.08	13.33 ± 5.77	11.67 ± 0.58	16.67 ± 7.64	8.67 ± 7.77
*Aspergillus ochraceus*	24.33 ± 6.03	16.66 ± 4.73	15.33 ± 2.89	20.00 ± 7.00	15.67 ± 3.79	19.33 ± 6.66
*Fusarium oxysporum*	ND	ND	ND	16.00 ± 3.46	ND	ND
*Fusarium* spp.	14.33 ± 1.15	11.00 ± 9.64	9.33 ± 8.14	11.67 ± 10.41	ND	17.33 ± 3.06
*Penicillium* spp.	17.33 ± 4.04	17.67 ± 2.08	21.33 ± 2.31	19.00 ± 1.73	19.00 ± 8.18	12.33 ± 11.23

Results are mean ± SD (*n* = 3); ND: Not detected.

Extracts 1, 2, and 3: Isolate RQ22 (*Bacillus thuringiensis*).

Extracts 4, 5, and 6: Isolate RQ13 (*Bacillus cereus*).

Extracts 1 and 4: Tryptic soy broth; Extracts 2 and 5: Rice medium; Extracts 3 and 6: Rice yeast medium.

Data in Table 3 clearly showed that extracts No. 2 and 5 of bacteria grown on rice medium showed higher antifungal activity against *A. flavus*, *A. niger*, and *Penicillium* spp. On the other hand, extracts No. 1 and 4 of bacteria grown on tryptic soy broth showed antifungal activity against *A. flavus* and *A. ochraceus*. Results showed that extracts No. 3 and 6 of bacteria grown on rice yeast extract medium showed low antifungal activity.

### Volatile organic compounds (VOCs) in bioactive metabolites

Twelve VOCs, representing 99.96%, were identified in extract No. 4 (Table 4, Figure S1), whereas the major components were butanoic acid, 2-methyl (37.64%) (Figure S2), iso-valeric acid (21.62%) (Figure S3), and propanoic acid, 2-methyl (19.13%) (Figure S4). Seven VOCs, representing 100%, were identified in extract No. 5 (Table 5, Figure S5), whereas the major components were 9, 12-Octadecadienoic acid (Z,Z)—(81.29%) (Figure S6). Hexadecanoic acid (7.75%) and 3, 6-Octadecadiynoic acid methyl ester (4.86%) were detected at a lower percentage. It could be observed that extract No. 5 contained high amounts of 9, 12-Octadecadienoic acid (Z,Z) -.

**Table 4:** Volatile organic compounds of extract No. 4

Peak No	RT (min.)	Area sum (%)	Chemical formula	Name	*m/z*	M^+^
1	7.11	19.13	C_4_H_8_O_2_	Propanoic acid, 2-methyl-^b^	43.1	88.1
2	7.29	5.32	C_4_H_8_O_2_	Butanoic acid^b^	41.1	108.0
3	11.49	21.62	C_5_H_10_O_2_	Iso-valeric acid	60.0	87.0
4	11.63	37.64	C_5_H_10_O_2_	Butanoic acid, 2-methyl-	74.0	87.0
5	14.44	0.72	C_4_H_8_O_2_S	Propanoic acid, 3-(methylthio)-	32.0	120.0
6	18.19	11.00	C_8_H_8_O_2_	Benzene acetic acid^a^	91.1	136.0
7	18.44	0.46	C_8_H_10_	Spiro[bicyclo[3.1.0]hexane-2,1'-cyclopropan]-3-ene	91.0	136.0
8	19.81	0.62	C_7_H_7_NO_2_	Beta-Methyl iso-nicotinic acid	91.0	137.0
9	24.40	1.31	C_10_H_16_N_2_O_2_	Cyclo (L-prolyl-L-valine)	154.1	185.1
10	25.18	1.07	C_11_H_18_N_2_O_2_	Pyrrolo [1,2-a] pyrazine-1,4-dione, hexahydro-3-(2-methyl propyl)-	154.1	195.0
11	26.08	0.54	C_16_H_32_O_2_	Hexadecanoic acid	41.1	256.2
12	29.00	0.56	C_14_H_16_N_2_O_2_	3-Benzyl-1,4-diaza-2,5-dioxobicyclo[4.3.0]nonane	125.1	244.1

RT, Retention time.

^a^Anticancer; ^b^Antioxidant; the activity of the chemical compounds is obtained from the National Center for Biotechnology Information.

**Table 5:** Volatile organic compounds of extract No. 5

Peak No	RT (min.)	Area sum (%)	Chemical formula	Name	*m/z*	M^+^
1	6.915	4.86	C_19_H_30_O_2_	3,6-Octadecadiynoic acid, methyl ester	43.0	178.1
2	8.085	1.64	C_5_H_9_NS	Isobutyl isothiocyanate	43.0	142.0
3	20.11	1.15	C_9_H_9_N	Benzene propanenitrile	32.0	162.0
4	25.558	7.75	C_16_H_32_O_2_	Hexadecanoic acid	41.1	256.2
5	27.381	81.29	C_18_H_32_O_2_	9,12-Octadecadienoic acid (Z,Z)^a^	41.1	280.3
6	35.18	2.41	C_29_H_50_O	Stigmast-5-en-3-ol, (3.beta.,24S)-	43.1	414.5
7	35.565	0.9	C_18_H_26_O	1,3-Bis-(2-cyclopropyl,2-methyl cyclopropyl)-but-2-en-1-one	32.0	207

RT, Retention time.

The activity of the chemical compounds is obtained from the National Center for Biotechnology Information.

^a^Antiviral.

### Polyphenols in bioactive metabolites

The ability of the two extracts No. 4 and 5 to produce polyphenols was investigated. Nine phenolic acids were detected (Table 6, Figure S7) in extract No. 4, whereas cinnamic acid and syringic acid were the most abundant phenolic acids at concentrations of 19.57 and 15.18 µg/g, respectively. Extract No. 4 reflected a distinguished content of flavonoids, with the detection of three compounds (Table 6). Daidzein and naringenin were detected at 24.10 and 14.38 µg/g, respectively. On the other hand, rutin was detected in relatively low concentrations recording 0.01 µg/g.

**Table 6:** Contents of phenolic acids and flavonoids detected in bioactive metabolites extracted from *Bacillus* spp.

Compounds	RT (min.)	Phenolic acid (µg/g)	Compounds	RT (min.)	Flavonoids (µg/g)
	Extract No. 4				
Gallic acid	3.86	0.15	Rutin	9.69	0.01
3.4-Dihydroxybenzoic acid^a^	5.75	1.76	Daidzein	12.89	24.10
Chlorogenic acid	7.33	0.09	Naringenin^a^	14.94	14.38
Syringic acid^a^	8.4	15.18	–		–
Coumaric acid	9.53	4.62	–		–
Vanillin^a^	9.57	2.67	–		–
Ellagic acid	9.90	0.03	–		–
Ferulic acid^a,b^	10.23	3.55	–		–
Cinnamic acid^a^	14.11	19.57	–		–

	Extract No. 5				
Gallic acid	3.83	10.49	Rutin	9.68	0.02
3.4-Dihydroxybenzoic acid^a^	5.73	560.22	Daidzein	12.90	1.60
Caffeic acid^a,b,c,d^	8.03	26.42	Naringenin^a^	14.96	9.22
Syringic acid^a^	8.36	20.88	–		–
Coumaric acid	9.52	50.92	–		–
Vanillin^b^	9.56	20.01	–		–
Ferulic acid^a,b^	10.23	40.06	–		–
Quercetin	13.53	0.03	–		–
Cinnamic acid^a^	14.12	57.33	–		–

The activity of the chemical compounds is obtained from the National Center for Biotechnology Information.

RT, Retention time.

^a^Antioxidant; ^b^Anticancer; ^c^Antiviral; ^d^Antibacterial.

Nine phenolic acids have been detected (Table 6, Figure S8) in extract No. 5, whereas 3, 4-dihydroxybenzoic acid was the most abundant phenolic acid at a concentration of 560.22 µg/g. Cinnamic acid and coumaric acid were also detected at high concentrations recording 57.33 and 50.92 µg/g, respectively. A distinguished content of flavonoids was also detected in extract No. 5, with three compounds (Table 6), whereas naringenin was detected at a concentration of 9.22 µg/g. On the other hand, daidzein and rutin were detected at lower concentrations recording 1.60 and 0.02 µg/g, respectively.

### Scanning electron microscope (SEM)

**Figure 3:**
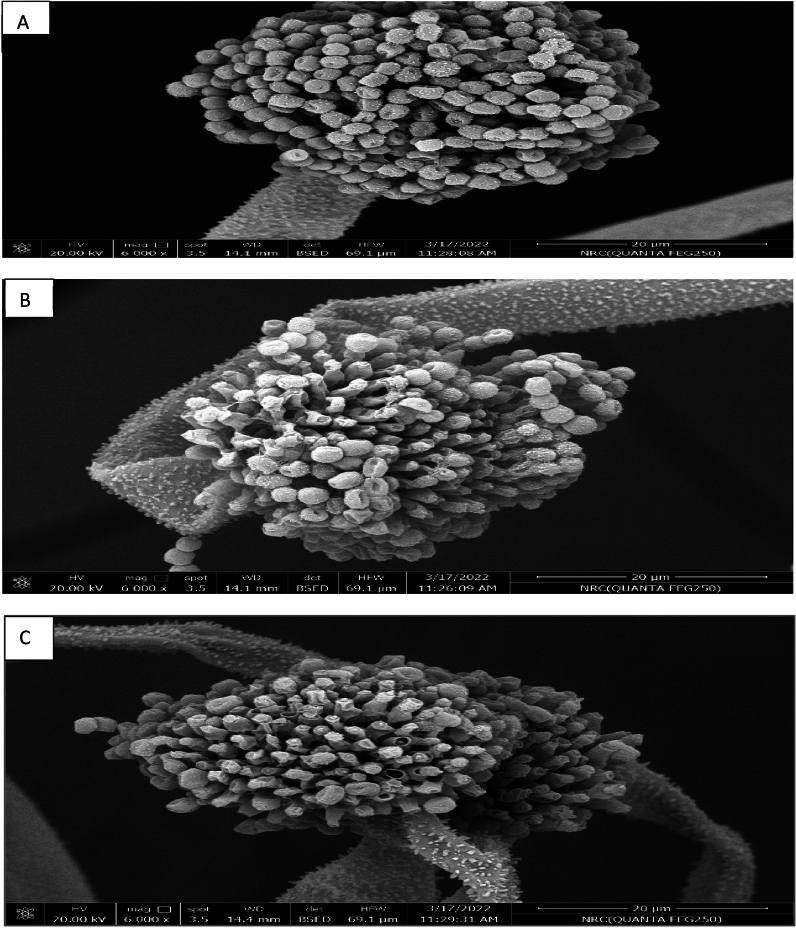
Scanning electron microscope of (**A**) untreated *A. flavus* (control) showing typical conidia and conidiospores; (**B**) *A. flavus* after treatment with extract No. 4 (extracted from *Bacillus cereus* grown on tryptic soy broth) showing loss and mutation of fungal conidiospores; and (**C**) *A. flavus* after treatment with extract No. 5 (extracted from *Bacillus cereus* grown on rice medium) showing loss and mutation of fungal conidiospores.

The effect of extracts No. 4 and 5 on *A. flavus* structure was determined using a SEM. Figure 3A shows typical conidia and conidiospores of untreated *A. flavus*. The effect of extract No. 4 on *A. flavus* spores followed with less deformation but also caused the loss of some fungal spores (Fig. 3B). On the other hand, extract No. 5 had a similar effect on the conidiophores and conidiospores of *A. flavus* (Fig. 3C).

### Antifungal and antiaflatoxigenic activity of bioactive metabolites

The effect of different concentrations of extracts No. 4 and 5 on the fungal growth of *A. flavus* after 10 days of incubation was studied (Table 7). Results revealed that extracts No. 4 and 5 at 9 mg/mL concentrations reduced fungal growth by 57.32% and 35.98%, respectively. On the other hand, data indicated that different concentrations of extract No. 4 showed variable degrees of antifungal activity, whereas extract No. 5 at concentrations 1 and 3 mg/mL stimulated fungal growth. Inhibition of fungal growth occurred by 21.70%, 30.66%, and 35.98% at concentrations 5, 7, and 9 mg/mL, respectively. It was also noticed that the inhibition of fungal growth increased by increasing the concentration of extracts.

**Table 7:** Effect of bioactive metabolites extracted from *Bacillu*s spp. on *A. flavus* growth and aflatoxin production.

Treatment	Concentration (mg/mL)	Dry mycelium weight		Aflatoxin production (ng/g)							
		mg/50 mL medium	%*	AFB_1_	%*	AFB_2_	%*	AFG_1_	%*	AFG_2_	%*
Control	–	137.3		1,935,723.75		21,505.4		3421.84		6456.91	
Extract No. 4	1	116.9	14.86%	9067.93	99.53%	69.36	99.68%	ND	100%	4645.36	28.06%
	3	101.8	25.86%	901.31	99.95%	14.37	99.93%	ND	100%	712.45	88.97%
	5	101.3	26.22%	349.62	99.98%	13.86	99.94%	ND	100%	103.19	98.40%
	7	96.8	29.50%	442.55	99.98%	ND	100%	ND	100%	27.08	99.58%
	9	58.6	57.32%	337.75	99.99%	ND	100%	ND	100%	63.86	99.01%
Extract No. 5	1	187.2	–	321,109.98	83.41%	1992.68	90.73%	550.98	83.90%	1877.69	70.92%
	3	174.2	–	10,612.26	99.45%	23.42	99.89%	318.72	90.69%	867.05	86.57%
	5	107.5	21.70%	542.69	99.97%	103.55	99.52%	259.04	92.43%	564.99	91.25%
	7	95.2	30.66%	457.12	99.98%	ND	100%	146.34	95.72%	144.14	97.77%
	9	87.9	35.98%	504.64	99.97%	ND	100%	350.24	89.76%	ND	100%

Values of dry mycelium weight were obtained by weighing mycelium mats.

*Percentage of inhibition.

The effect of different concentrations of extracts No. 4 and 5 on aflatoxin production was investigated (Table 7). The data indicated that extract No. 4 at a 1 mg/mL concentration, reduced AFB_1,_ AFB_2_, and AFG_2_ by 99.53%, 99.68%, and 100%, respectively. At concentrations of 7 and 9 mg/mL AFB_2_ production was completely prevented. On the other hand, AFG_1_ production was completely prevented at concentrations 1, 3, 5, 7, and 9 mg/mL. Results also revealed that at a concentration of 9 mg/mL, an increase in AFG_2_ production was observed.

In studying the effect of extract No. 5 on aflatoxin production, results indicated that AFB_1_ was reduced by 83.41% at a concentration of 1 mg/mL, and AFB_1_ production was continuously reduced by increasing the concentration of the extract. For AFB_2_ production, the extract at a concentration of 1 mg/mL reduced AFB_2_ by 90.73%, whereas at concentrations 7 and 9 mg/mL, AFB_2_ was not detected. Data also showed that the extract, at a concentration of 1 mg/mL, reduced AFG_1_ by 83.90%, and the reduction increased by increasing the extract concentration to 9 mg/mL to reach 95.70%. On the other hand, AFG_2_ was reduced by 70.92% at a concentration of 1 mg/mL, whereas the reduction increased by increasing the concentration to 9 mg/mL to reach 100%.

### Brine shrimp lethality bioassay

**Figure 4:**
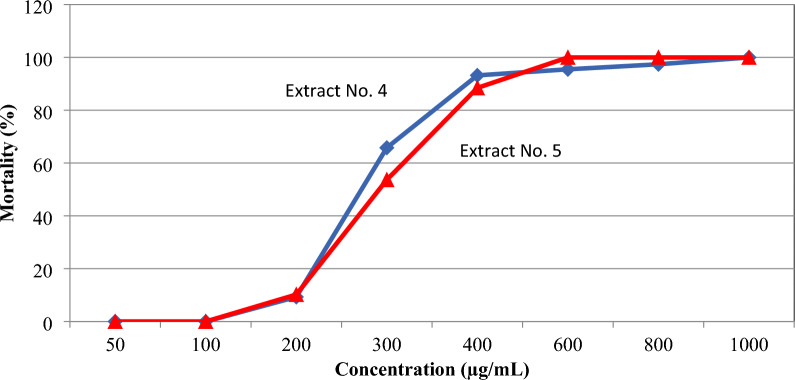
Effects of bioactive metabolites on *A. salina* mortality.

The effect of extracts No. 4 and 5 on the mortality percentage values of the *A. salina* are presented in Fig. 4. The toxicity test results revealed the highest percentage of mortality for larvae, which was recorded at a concentration of 600 µg/mL for extract No. 5. On the other hand, extract No. 4 showed the lowest percentage of mortality for larvae, which was recorded at a concentration of 1000 µg/mL. The LC_50_ value was obtained based on the mortality of *A. salina* larvae induced by the extracts, with extract No. 5 showing the highest LC_50_ at 300 µg/mL, followed by extract No. 4 at 275 µg/mL.

### Cytotoxicity of human cell line

**Figure 5:**
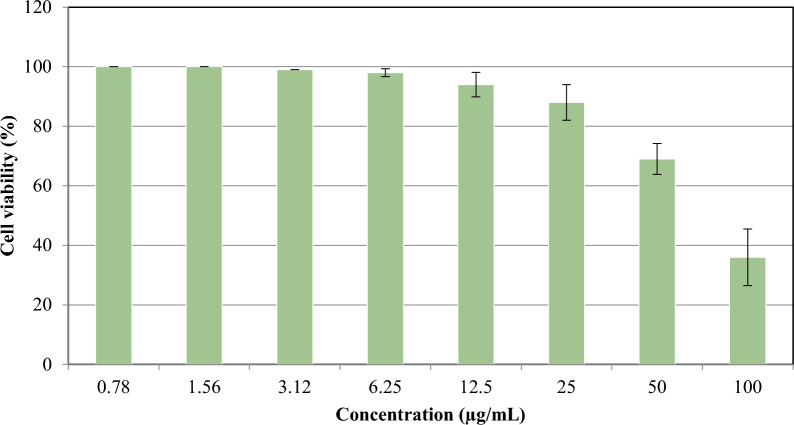
Effect of different concentrations of extract No. 5 on HepG2 cell line viability.

Extract No. 4 showed no anticancer activity; nevertheless, extract No. 5 produced cell toxicity in a concentration-dependent manner (Fig. 5). The IC_50_ values were used to express anti-proliferative activity, whereas lower IC_50_ values indicated more significant cell growth-inhibiting activity. Data showed that extract No. 5 had an IC_50_ value of 79.4 µg/mL, whereas doxorubicin, which was used as a positive control, had an IC_50_ value of 21.6 µg/mL (Figure S9). It was observed that the extracts tested were not active as doxorubicin.

## Discussion

One of the most vital points of this research is the screening of novel microorganisms for antifungal activity. The CFS of thirty-six bacterial isolates were tested for their antifungal activity in this study, whereas the CFS of the following bacterial isolates No. RQ2, RQ7, RQ13, RQ15, and RQ22, were the most promising inhibiting *Fusarium* spp. to a varying degree. Similarly, several authors established the ability of *Bacillus* strains to successfully biocontrol *Fusarium* strains^32–34^. In another study, eight newly grown *Bacillus* isolates (GB31, GB41X, XJ11, XG11, B. sub2, XJ2, XJ8, and XI4) effectively inhibited the growth of more than two *F. graminearum* strains in vitro^35^.

*Aspergillus flavus* is a microscopic saprophyte fungus that is both pathogenic and toxic and is common in nature and reduces the quality and safety of raw materials and products. The CFS of the bacterial isolates No. RQ1, RQ2, RQ5, RQ15, RQ16, and RQ22 inhibited *A. flavus.* In agreement, Idiyatov et al.^36^ stated that isolates EFS10, EFS14, and EFS17 from the *B. subtilis* species had the most pronounced antifungal activity against *A. flavus.* On the other hand, *A. parasiticus* was inhibited by CFS of the bacterial isolates No. RQ1, RQ2, RQ7, RQ13, RQ15, RQ16, and RQ22. Siahmoshteh et al.^15^ revealed that both *B. subtilis* and *B. amyloliquefaciens* demonstrated significant antifungal activity against a wide range of filamentous fungi, and they were successful in suppressing the growth of *A. parasiticus* by up to 92%.

Also, the CFS of the bacterial isolates No. RQ8 and RQ13 only inhibited *A. ochraceus*. In agreement, Zhao et al.^37^ reported that the antifungal activity of the *B. subtilis* CW14 supernatants was the best, inhibiting mycelial growth and spore germination of *A. ochraceus*. The antifungal activity of *Bacillus* isolates could be attributed to the generation of antifungal chemicals that influence the morphological development of fungal structures, such as hyphae growth, morphology, and spore germination^15^.

The bacterial isolates showing high antifungal activity were identified using biochemical parameters and MALDI-TOF MS analysis. The MALDI-TOF MS has been used as a quick, high-throughput bacterial identification technology in diagnostic microbiology laboratories^38^. However, this identification technique's efficacy depends on the reference strains obtained in the mass spectral database^39^. As previously stated, spectral analysis with MALDI-TOF MS can complete identifications as high as 16S rRNA sequences due to its ability to identify at the species level^40,41^.

The bacterial isolates No. RQ13 and RQ22 which showed higher antifungal activity were thus selected to extract and characterize their bioactive secondary metabolites, whereas VOCs were identified using gas chromatography-mass spectrometry (GC/MS). Our results revealed twelve VOCs were detected in extract No. 4, whereas seven VOCs were detected in extract No.5. Our results were considered lower than those reported by Gao et al.^42^, who isolated and identified *B. subtilis* CF-3, and later found that during the fermentation process, a total of 74 potential VOCs were identified^43^. Similar observations were reported by Rajaofera et al.^44^, who identified nineteen different VOCs from *B. atrophaeus* HAB-5. In a similar study, twenty-nine VOCs were identified from *B. methylotrophicus* BCN2. Meanwhile, thirty VOCS were identified from *B. thuringiensis* BCN10^45^. Furthermore, the results agree with Zhang et al.^46^, who showed that *B. subtilis* might produce antibiotics, antifungal proteins, and a variety of VOCs^47^.

The main components in extract No. 4 were butanoic acid, 2-methyl (37.64%), and iso-valeric acid (21.62%). On the contrary, Sadiq and Jamil^48^ revealed that the extract of intracellular compounds produced by *B. cereus* showed the presence of VOCs such as toluene, acetic acid butyl ester, 2-Pentanol acetate, and propanoic acid. The VOC hexadecanoic acid was also detected at low concentrations. Similar observations were reported by Rajaofera et al.^44^, who noted that hexadecanoic acid is one of the compounds produced by *B. atrophaeus* strain HAB-5. The most critical components identified in extract No. 5, were 9, 12-Octadecadienoic acid (Z,Z)—(81.29%), and hexadecanoic acid (7.75%). Similar results were reported by Jangir et al.^49^, who identified the following compounds; namely 9,12-octadecadienoic acid (Z,Z)-, and hexadecanoic acid from *B. subtilis*.

One of the most significant antifungal compounds detected at a high percentage in extract No. 4 was butanoic acid 2-methyl-^50^. Similar observations were reported by Xu et al.^51^, who stated that VOCs 2 methylbutanoic acid and iso-valeric acid released by *B. tequilensis* XK29 inhibited the growth of *Ceratocystis fimbriata*. On the other hand, the VOC 9, 12-octadecadienoic acid (Z,Z) detected at a high percentage in extract No. 5 was confirmed to be an excellent antifungal agent^52^.

Polyphenols are the most abundant type of natural antioxidant, flavonoids, hydrolyzable and condensed tannins, phenolic acids, stilbenes, lignans, and phenolic aldehydes^53^. The LC/MS detected nine phenolic acids in extract No. 4, whereas cinnamic and syringic acids were the most abundant. Extract No. 4 reflected a distinguished content of flavonoids, with the detection of daidzein, naringenin, and rutin. In agreement, Hassan et al.^54^ found syringic acid, among other phenolic acids. produced by several *Bacillus* spp., whereas rutin was the only flavonoid produced.

Nine phenolic acids in extract No. 5 were detected. The phenolic acids 3, 4-dihydroxybenzoic acid, and cinnamic acid were the most abundant. On the other hand, a distinguished content of flavonoids was also detected in extract No. 5, with the detection of naringenin, daidzein, and rutin. Similar observations were reported by Zhao et al.^55^, who successfully applied to an in vitro study in which catechin/epicatechin-broth samples were anaerobically fermented with gut microbes obtained from healthy human donors, and all bacteria used demonstrated outstanding ability in metabolizing grape polyphenols, whereas 3, 4‑dihydroxybenzoic acid was detected among other phenolic acids.

Recently, phenolic compounds have garnered much attention due to their antioxidant activity; additionally, these compounds have been reported to be potential candidates in lowering cardiovascular diseases^56^ and anti-carcinogenic activities, antiallergenic, anti-atherogenic, anti-inflammatory, antimicrobial, and antithrombotic effects^57^. Phenolics are gaining popularity in the food sector because they slow the oxidative breakdown of lipids, improving food quality and nutritional content^58,59^.

In the setting of developing antibiotic resistance, there is a need for active chemicals that inhibit disease propagation, infection, and virulence. The food production industry also relies heavily on developing novel food preservatives that are less damaging to human and environmental health. This prospective sector of microbial VOC application has yet to be investigated^60^.

Results showed that extracts No. 4 and 5 extracted from *B. cereus* showed higher antifungal activity against various fungal species. Hathout et al.^61^ reported similar results and found that *Bacillus* extract inhibited the growth of *Candida albicans* and *Aspergillus niger*. Miljaković et al.^62^ reported similar observations, stating that *B. cereus* MH778713's antifungal activity could be linked to the generation of diffusible metabolites and hydrolyzing enzymes. Although only one *Bacillus* extract had antifungal activity on *F. oxysporum*, Ramírez et al.^63^ revealed that *B. cereus* MH778713 VOCs reduced the mycelial radial growth of *F. oxysporum* by 38%.

Later, different concentrations of extract No. 4 and 5 were studied on *A. flavus* fungal growth*,* and aflatoxin production after 10 days of incubation. Data showed extracts No. 4 and 5 reduced fungal growth. It was also noticed that increasing the concentration of extracts increased the inhibition of fungal growth. Similar results were reported by Siahmoshteh et al.^15^, who revealed that culture filtrates of soil strains of *B. subtilis* and *B. amyloliquefaciens* showed antifungal activity against *A. parasiticus* NRRL2999. In agreement, Khan et al.^34^ reported that 100 µg/mL of 1-butanol extract of d *B. subtilis* 30VD-1 cell-free culture filtrate caused about 40% inhibition of *Fusarium* spp. The antifungal activity of extracts No. 4 and 5 might be due to bioactive secondary metabolites such as VOCs and polyphenols, known as antimicrobial compounds.

Data also showed that extracts No. 4 and 5 at a 9 mg/mL concentration reduced AFB_1_ production by 99.99% and 99.97%, respectively. On the other hand, extract No. 4 at a concentration of 9 mg/mL completely prevented AFG_1_ and reduced AFG_2_ output by 99.01%, whereas extract No.5 reduced AFG_1_ by 89.76% and prevented AFG_2_ production. In experimental animals, Abdel-Wahhab et al.^64^ revealed that *Bacillus* extract at the tested doses improved all biochemical parameters and the histological image in rats that received AFB_1_. In vitro, Pereyra et al.^65^ indicated that *Bacillus* spp. reduced AFB_1_ production by *A. parasiticus*, whereas extracellular metabolites extracted from the *Bacillus* species inhibited AFB_1_ production to non-detectable levels. In another study, the CFS of *B. licheniformis* CFR1 was able to degrade AFB_1_ more efficiently than the cell lysate^66^. Recently, *L. rhamnosus* bioactive secondary metabolites at a 9 mg/mL concentration reduced AFB_1_ production by 99.98%^30^.

The brine shrimp lethality bioassay was initially employed as a simple test to determine toxicity; nevertheless, it is not a specific test. The brine shrimp lethality bioassay is a quick, inexpensive, and straightforward method for assessing the biological activities of extracts and is considered an initial screening method used to evaluate bioactive compounds or compounds assumed suitable as anticancer drugs. The brine shrimp lethality bioassay has been widely used as a preliminary screening of bioactive metabolites to assess their toxicity to *A. salina*, which could potentially indicate the potential cytotoxic qualities of the test materials^67^. Extract No. 5 showed the highest LC_50,_ recording 300 µg/mL, followed by extract No. 4, recording an LC_50_ of 275 µg/mL.

The current investigation revealed that the degree of mortality of *A. salina* larvae was proportional to the concentration of the extracts. That could be because the higher the concentration, the more potent bioactive contents can be produced. As there is a correlation between cytotoxicity and activity against *A. salina* nauplii, the cytotoxic effects of the extracts were chosen for subsequent cell line assay^68^. The extracts showing toxicity against *A. salina* were studied against HepG2. Results revealed that extract No. 4, which displayed low toxicity against *A. salina*, showed no anticancer activity. On the other hand, extract No. 5, which showed high toxicity against *A. salina*, was effective against HepG2 cells by 64.3% at a concentration of 100 µg/mL and recorded an IC_50_ of 79.4 µg/mL. Similar results were reported by Haneen et al.^69^, who studied the effect of *B. cereus* and *B. subtilis* extracts against human breast adenocarcinoma (MCF-7) and found that at a concentration of 100 µg/mL, the extracts were effective against MCF-7 cells for both *B. cereus* (48.8%) and *B. subtilis* (63.8%). In agreement, Ganguly et al.^70^ stated that the MTT experiment demonstrated significant cytotoxic activity against MCF-7 with an IC_50_ value of 46.64 µg/mL.

This study indicated that bioactive metabolites derived from *Bacillus cereus* dramatically inhibited AFs production showing a high potential for managing AFs contamination in the food and feed industries. Cytotoxicity experiments using the brine shrimp lethality assay and the HepG2 cell line revealed that *Bacillus cereus* bioactive metabolites had tolerable toxicity levels, thus indicating that the bioactive metabolites are safe to use in applications involving human or animal food items.

The various biological activities of bacterial bioactive metabolites make them useful in a variety of industrial applications, as they could be utilized to manufacture antibiotics, antifungals, anticancer agents, and antivirals^71^. Bacterial bioactive metabolites could be used in wastewater treatment to eliminate contaminants^72^. They are also essential in the production of bio-fertilizers, biofuels, cosmetics, and biopolymers^72^. Bacterial bioactive metabolites could have considerable therapeutic potential because they include antibacterial, antifungal, antiviral, and antioxidant properties, which are vital in the face of growing drug-resistant microbial infections^71^.

## Materials and methods

### Chemicals

Ethyl acetate and chloroform HPLC grade were purchased from Merck KGaA (Darmstadt, Germany), whereas dimethyl sulfoxide (DMSO) was purchased from Research Lab Fine Chem Industries (Mumbai, 400 002, India). Sodium sulfate anhydrous and yeast extract were purchased from Loba Chemie (Mumbai 400 005, India). The mediums potato dextrose agar, nutrient agar, and tryptic soy broth were purchased from Neogen (Lansing, MI 48912, USA).

### Sampling

Rice grains (1 kg) were gathered from retail stores in Egypt's Qalyubia governorate, and they were transported to the Food Toxicology and Contaminants lab and kept in polythene bags refrigerated (< 10 °C) until examination.

### Microorganisms

The genome sequence of the aflatoxin-producing *Aspergillus flavus* utilized in this work was submitted to the GenBank database as *A. flavus* AAM2020 (Accession No. OP942201) and was isolated from Egyptian maize samples^73^. From several grains in Egypt, the fungi *A. parasiticus*, *A. niger*, *A. ochraceus*, *Fusarium oxysporum*, *Fusarium* spp., and *Penicillium* spp. were isolated^74^.

### Isolation of endophytic bacteria

The rice grains were washed in running water for ten minutes and then washed two times with sterile distilled water for one minute; after that, they were immersed in 3% (v/v) sodium hypochlorite and 70% (v/v) ethanol for three minutes. The rice was washed thrice with sterilized distilled water for two minutes to finish cleaning. Sterilized rice grains were placed on sterilized filter paper before implanted on nutrient agar Petri dishes and incubated at 30 °C for 2 to 7 days^75^. Sub-culturing was employed to select and purify morphologically distinct colonies. Pure cultures were used to evaluate their antifungal activity.

### Antifungal activity of CFS of bacterial isolates

Endophytic bacterial isolates were tested for their antifungal activity using the agar well diffusion technique^76^. Isolated endophytic bacteria strains were cultured on tryptic soy broth for 24 h at 37 °C. Following incubation, the broth media were centrifuged (Thermo Fisher Scientific, USA) at 10,000 rpm for 10 min at 4 °C to collect the CFS, which was then filtered using a 0.2 mm sterile Millipore filter (Millex-GS, Millipore, USA). On Petri dishes, potato dextrose agar media was placed, and different fungal isolates (50 µL, 10^6^ spores/mL) were dispersed completely over the agar surface with a sterile cotton swab. Wells were cut with a sterile cork borer and each well-received 100 µL of CFS of bacterial isolates. The Petri dishes were incubated for 48 h at 28 °C, and the inhibition zones (mm) were measured to assess the antifungal activity. Biochemical analysis and MALDI-TOF were used to identify endophytic bacterial isolates with antifungal activity.

### Identification of bacterial isolates

The bacterial isolates showing antifungal activity were identified using biochemical parameters (Gram staining^77^ and oxidase test^78^) and MALDI-TOF MS. In brief, a loop of freshly bacterial cultures was placed in 300 µL of water and 900 µL of ethanol. The pellets were mixed with equivalent formic acid (70%) and acetonitrile (100%) after centrifugation for 2 min at 13,000 rpm. Using the MALDI-TOF MS technology, the supernatant (extracted bacterial proteins) was utilized for protein identification and profiling (Microflex LT, Bruker, Billerica, MA 01821, USA)^79^.

For identification, the sample (1 µL) was placed on a MALDI bio target plate and left to dry at room temperature. The dried sample spot was then covered with a (1 µL) matrix of α-cyanohydroxy cinnamic acid (CHCA) to enable the proteins in the sample to crystallize. The MALDI bio target plate was then put into the MALDI-TOF apparatus. The identification score (from 0 to 3) was then used to characterize the amount of mass spectral concordance with the database. The MBT Compass and Flex Analysis tools were used to analyze and process these data.

### Extraction of bioactive metabolites

The bacterial isolates No. RQ13 and RQ22 were grown on rice medium (100 g rice in 100 mL distilled water) and rice and yeast medium (100 g rice, 0.5 g yeast extract, in 100 mL distilled water). The rice mediums were incubated at 35 °C in static for 7 days. Simultaneously, the bacterial isolates No. RQ13 and RQ22 were cultured in tryptic soy broth and incubated at 35 °C with shaking at 120 rpm for 3 days^80^. After incubation, the broth media were centrifuged at 10,000 rpm (Thermo Fisher Scientific, USA) for 10 min at 4 °C to collect the CFS, which was then filtered through sterilized 0.22 µm pore-size filters (Millex-GS, Millipore, USA). Ethyl acetate was added to the CFS and rice medium in a 1:1 (v/v) ratio and vigorously agitated for a few minutes. The ethyl acetate mixtures were put into separating funnels and let to stand until the organic and aqueous phases separated, at which point the organic phase was collected and passed through anhydrous sodium sulfate. This procedure was performed three times before the organic phase was dried off using a rotary evaporator (Heidolph Instruments GmbH & Co. KG, Germany) to produce extracts^44^.

### Determination of antifungal activity of bioactive metabolites

The antifungal potential of the extracts was assessed using an agar well diffusion experiment, as reported by Salman et al.^81^. On Petri dishes, potato dextrose agar medium was applied, and various fungal isolates (100 µL, 10^6^ spores/mL) were dispersed equally across the agar surface with a sterile cotton swab, followed by wells cut with a sterile cork borer. Then, 100 µL of extracts No. 4 and 5 were applied to each well at a 5 mg/mL concentration. The Petri plates were incubated for 48 h at 28 °C, and the inhibition zones (mm) were measured to assess the antifungal activity.

### Determination of volatile organic compounds in bioactive metabolites

The gas chromatography/mass spectrometer (GC/MS) technology (Agilent Technologies, USA) was used to undertake a qualitative and quantitative analysis of volatile organic molecules. A gas chromatograph (7890B) and a mass spectrometer detector (5977A) were part of the GC/MS system. The GC was outfitted with an HP-5MS column (30 m × 0.25 mm internal diameter and 0.25 m film thickness). The carrier gas in the analyses was hydrogen, with a flow rate of 1 mL/min at a splitless injection volume of 1 µL and the following temperature program: 50 °C for 1 min; 5 °C/min rise to 100 °C and hold for 0 min; 10 °C/min rise to 300 °C and hold for 5 min. The injector and detector were held at temperatures of 250 °C and 260 °C, respectively. With a 6-min solvent delay, electron ionization (EI) at 70 eV produced mass spectra with a spectral range of m/z 50–550. Different components were found by comparing the spectrum fragmentation pattern to those in the Wiley and NIST Mass Spectral Library data.

### Determination of polyphenols in bioactive metabolites

Extracts were analyzed to assess polyphenols using liquid chromatography-electrospray ionization-tandem mass spectrometry (LC–ESI–MS/MS), with an Exion LC AC system for separation and a SCIEX Triple Quad 5500 + MS/MS system with electrospray ionization (ESI) for detection. A ZORBAX SB-C18 Column (4.6 X 100 mm, 1.8 µm) (Agilent Technologies, USA) was employed for the separation. Two eluents were used in the mobile phases: A (0.1% formic acid in water) and B (acetonitrile). The mobile phase was set at 2% B from 0 to 1 min, 2% B from 1 to 21 min, 60% B from 21 to 25 min, and 2% B from 25 to 28 min. The injection volume was 3 µL, and the flow rate was 0.8 µL/min. The following parameters were employed for MRM analysis of the chosen polyphenols in both positive and negative ionization modes: Ion spray voltages for the positive and negative modes were 4500 and -4500, respectively; 400 °C source temperature; 25 psi curtain gas; 55 psi ion source gases with a declustering potential of 50; 25 psi collision energy; and 10 psi collision energy spread.

### Evaluation of the effect of the bioactive metabolites on the microstructure of *Aspergillus flavus* using a SEM

The effect of extracts on *A. flavus* microstructure was investigated. Twenty-five milliliters of molten potato dextrose agar were put in Petri plates and permitted to solidify. A fungal spore suspension of *A. flavus* (100 µL, 10^6^ spores/mL) was distributed evenly over the agar surface, after which wells were cut using a sterile cork borer. Each well received 100 µL of extracts Nos. 4 and 5, and the plates were incubated at 28 °C for 5 days. Mycelium segments (1 cm^2^) were cut and deposited at room temperature in vials containing 3% glutaraldehyde in 0.05 M phosphate buffer (pH 6.8), as stated by Mims^82^ and Gong et al.^83^. An ethanol series was used after chemical fixation, culminating in total ethanol. Fungal cultures were freeze-dried after samples were dried in liquid carbon dioxide. The samples were put in Petri plates, and an unfilled portion of the plate was filled with a vial cap holding 4% osmium tetraoxide in water. Then, segments were coated with 20 to 30 nm of 60:40 gold palladium. All materials were examined in a 20.00 kV electron probe micro-analyzer SEM (Quanta FEG 205, FEI Company, Hillsboro, OR, USA).

### Effect of bioactive metabolites on fungal growth and aflatoxin production

The impact of extracts No. 4 and 5 on mycelial dry weight (MDW) and AFs production was determined using the method described by Roshan et al.^5^ with a few adjustments (using potato dextrose broth instead of sucrose malt-yeast extract-broth). The appropriate amounts of extracts were added to reach final concentrations of 1, 3, 5, 7, and 9 mg/mL of growth media to various flasks containing 20 mL of potato dextrose broth. Each flask was filled with a fungal spore solution (100 µL, 10^6^ spores/mL) from a 7-day-old culture of the aflatoxigenic isolate. For 10 days, cultures were incubated at 28 °C. After incubation, the culture medium was filtered (Whatman No. 1), and the mycelia were washed with water and dried in a hot air oven (110 °C, 12 h). In a separating funnel, the filtrate was extracted twice with 20 mL chloroform, and then the extract was passed through anhydrous sodium sulfate and evaporated to dryness. HPLC was used to identify aflatoxins. The percentage of inhibition was calculated using the following equation:

1 $$Percentage\;of\;inhibition \left( \% \right) = 1 - \frac{Treatment}{{Control}} \times 100$$

### Brine shrimp lethality bioassay

The in vivo mortality of extracts No. 4 and 5 were estimated using nauplii of the *A. salina*. Twenty-seven g of the commercially available salt was dissolved with 900 mL of distilled water to make artificial saltwater. The *A. salina* eggs were placed in a small commercial tank with artificial seawater for nauplii hatching and incubated for 48 h under a halogen lamp, which provided direct light and warmth.

Twenty mg of extracts No. 4 and 5 were diluted in 2 mL of ethyl acetate, and then concentrations of 50, 100, 200, 400, 600, 800, and 1000 g/mL were made. The tubes were allowed to dry out completely. Following that, each tube received 4.5 mL of artificial seawater, and ten nauplii were counted macroscopically and transferred to test tubes using the stem of a graduated Pasteur pipette against an illuminated backdrop. With artificial seawater, the final volume in each tube was adjusted to 5 mL after introducing the nauplii. Two separate counters counted and recorded the quantity of surviving nauplii in each tube after 24 h^84,85^. The experiment included three replicates for each treatment and 10 nauplii per replication. The LC_50_ values were calculated with 95% confidence intervals using data analysis and interpreted using the Reed-Muench technique. The Reed-Muench technique presupposes that an animal that survives a particular dose will likewise survive any lower dose and that an animal that dies at a specific dose would similarly die with any higher dose. Thus, throughout the range of doses investigated, information from any group may add to knowledge from other groups^86,87^.

### Cytotoxicity of human cell line

The impact of extracts No. 4 and 5 on HepG2 was investigated. At 37 °C and 5% CO_2_, cells were suspended in DMEM-F12 media supplemented with 1% antibiotic–antimycotic combination (10,000 U/mL potassium penicillin, 10,000 g/mL streptomycin sulfate, and 25 g/mL amphotericin B) and 1% L-glutamine.

Cell viability was determined using the mitochondrial-dependent reduction of yellow MTT (3-(4,5-dimethylthiazol-2-yl)-2,5-diphenyl tetrazolium bromide) to purple formazan^88^. Cells were cultivated for 10 days before being seeded at a density of 10 × 10^3^ cells/well in fresh complete growth medium on 96-well microtiter plates at 37 °C for 24 h in a water-jacketed carbon dioxide incubator (Sheldon, OR 97113, USA).

The cells were cultivated alone (control) or with different extract concentrations to obtain a final concentration of (0.78, 1.56, 3.125, 6.25, 12.50, 25.00, 50.00, and 100.00 µg/mL). After 48 h, the medium was sucked; 40ul MTT salt (2.5 µg/mL) was added to each well and incubated for another four hours at 37 °C with 5% CO_2_. Each well received 200 µL of deionized water containing 10% sodium dodecyl sulfate (SDS) and was incubated overnight at 37 °C to terminate the reaction and dissolve the generated crystals. A positive control of Adriamycin (Doxorubicin), a recognized cytotoxic natural chemical with a 100% death rate under identical circumstances, was used at a 100 µg/mL concentration.

Using a microplate multi-well reader (Bio-Rad Laboratories Inc., model 3350, Hercules, California, USA), absorbance was measured at 595 nm and a reference wavelength of 620 nm. The extracts were dissolved in DMSO, with a final concentration of less than 0.2% on the cells. The following equation was used to compute the percentage of viability:

2 $$Percentage\;of\;viability = \left( {\frac{Reading\;of\;extract}{{Reading\;of\;negative\;control}} - 1} \right) \times 100$$

The degree of selectivity of the synthesized compounds is stated in the current study as SI = IC_50_ of the pure compound in a normal cell line/IC_50_ of the same pure compound in a cancer cell line, where IC_50_ is the concentration necessary to kill 50% of the cell population. In vitro*,* bioassay on human tumor cell line test was conducted by the Bioassay-cell culture Laboratory, National Research Centre, Cairo, Egypt.

### Statistical analysis

The statistical analyses were conducted using the SPSS 26 (IBM, USA) software. The studies were carried out in triplicate, and the differences between control and treatment groups were assessed using the Student's t-test, while the differences across the groups were examined using the one-way ANOVA test. The significance threshold was chosen at *P* ≤ 0.05.

## Conclusion

The isolated and identified *Bacillus* species showed variable degrees of antifungal activity. The bioactive metabolites extracted from these *Bacillus* species produced volatile organic compounds and polyphenols and exhibited antifungal and antiaflatoxigenic activity. These bioactive metabolites induced toxicity against *A. salina* and against hepatocellular carcinoma. This study is considered the first to report *Bacillus* bioactive metabolites' ability to reduce and prevent aflatoxin production.

### Supplementary Information

Supplementary Figures.

## Data Availability

The datasets generated during the current study are available from the corresponding author upon reasonable request.
