# Supplementary material for: Antifungal, antiaflatoxigenic, and cytotoxic properties of bioactive secondary metabolites derived from Bacillus species
Source: Sci Rep. 2024 Jul 18;14:16590. doi: 10.1038/s41598-024-66700-y (PMC11258281; doi:10.1038/s41598-024-66700-y)
Supplement: Supplementary file 1 — Supplementary Figures. [file 41598_2024_66700_MOESM1_ESM.docx]

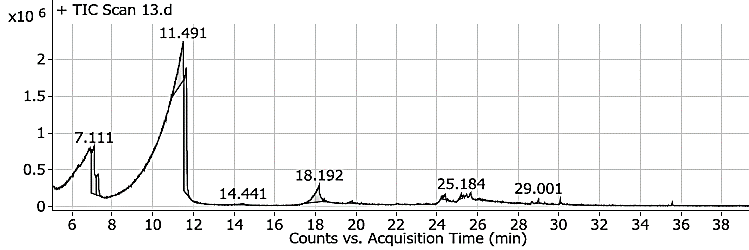


**Figure (S1): The GC/MS chromatogram of extract No. 4**


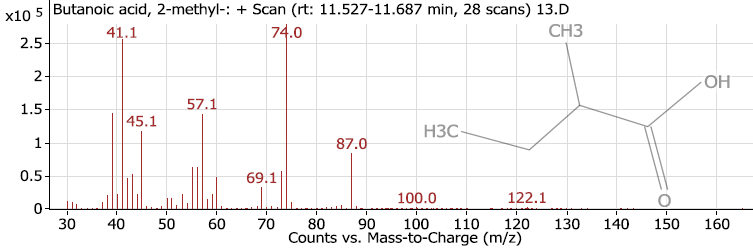


**Figure (S2): Chemical structure and GC/MS spectrum of Butanoic acid, 2-methyl** **detected in extract No. 4**


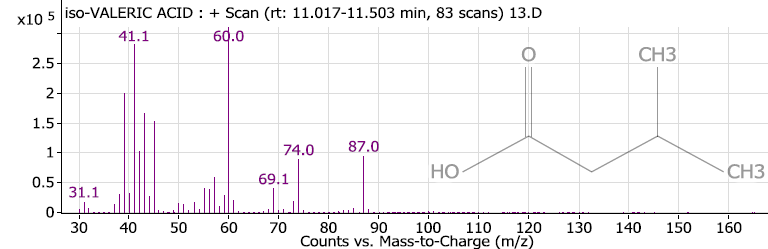


**Figure (S3): Chemical structure and GC-MS spectrum of iso-valeric acid detected in extract No. 4**


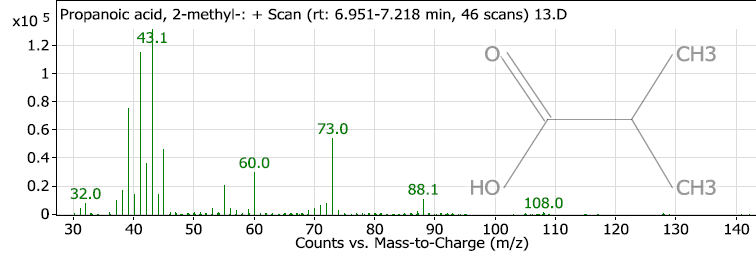


**Figure (S4): Chemical structure and GC/MS spectrum of propionic acid, 2-methyl** **detected in extract No. 4**


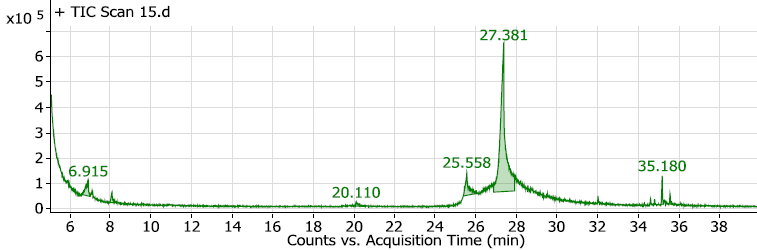


**Figure (S5): The GC/MS chromatograms of extract No. 5**


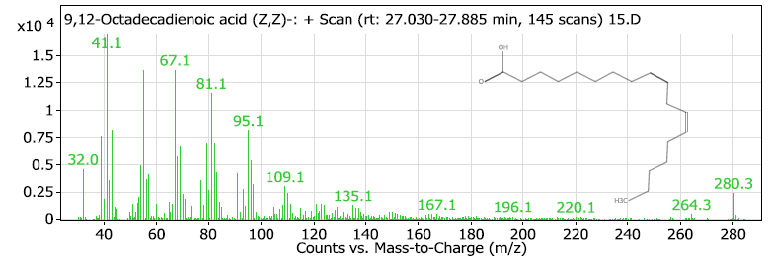


**Figure (S6): Chemical structure and GC/MS spectrum of 9, 12 Octadecadienoic acid (Z, Z) detected in extract No. 5**


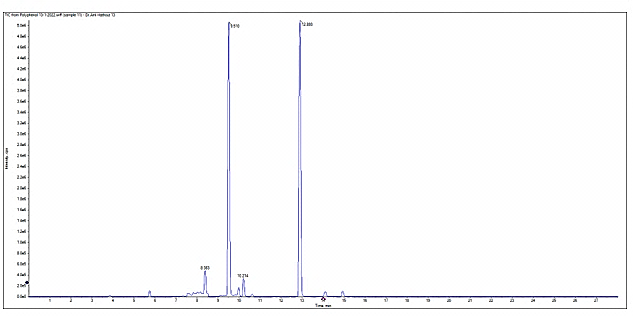


**Figure (S7): The LC/MS chromatogram of extract No. 4**


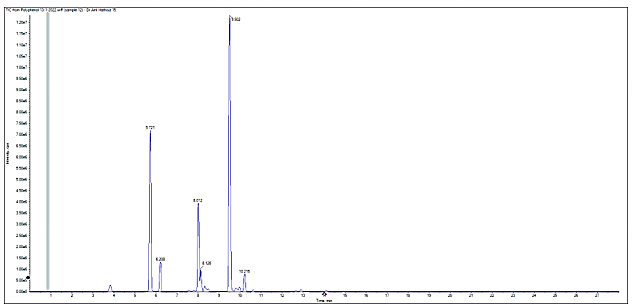


**Figure (S8): The LC/MS chromatogram of extract No. 5**

**Figure (S9): The IC_50_ of extract No. 5 compared to the positive control doxorubicin.**
